# Changes in Diffuse Tensor Imaging and Therapeutic Effect of Repetitive Transcranial Magnetic Stimulation in Traumatic Brain Injury with Central Pain

**DOI:** 10.3390/brainsci10120929

**Published:** 2020-12-02

**Authors:** Dong-Ha Kang, Gi-Wook Kim

**Affiliations:** 1 Department of Physical Medicine & Rehabilitation, Jeonbuk National University Medical School, Jeonju 54907, Korea; fanta0915@gmail.com; 2 Research Institute of Clinical Medicine of Jeonbuk National University—Biomedical Research Institute of Jeonbuk National University Hospital, Jeonju 54907, Korea

**Keywords:** traumatic brain injury, neuropathic pain, therapeutics, diagnosis

## Abstract

Post-trauma chronic pain characterized by central pain is a symptom following traumatic brain injury (TBI). Studies on the effect of repetitive transcranial magnetic stimulation (rTMS) on central pain and the association between central pain and spinothalamic tract (STT) have been reported, but few studies have examined the effect of rTMS in patients with mild TBI with central pain through changes in diffusion tensor imaging (DTI)-based metrics of STT before and after rTMS. This case series aimed to investigate the therapeutic effect of rTMS in TBI with central pain and the changes in diffusion tensor imaging (DTI)-based metrics of the spinothalamic tract (STT) before and after rTMS. This study included four patients who complained of severe pain in the left or right side of the body below the neck area after a car accident. We performed numeric rating scale (NRS), bedside sensory examination, electrodiagnostic study, and DTI-based metrics of the STT before and after rTMS. According to the guidelines of the diagnosis and grading for neuropathic pain, all patients had neuropathic pain corresponding to “probable grade.” In all patients, rTMS was applied to the contralateral M1 cortex on the more painful side. There were no medication changes and other interventions during the rTMS. After rTMS, NRS decreased, bed sensory testing improved, and DTI-based STT metrics increased in all patients compared to before rTMS.

## 1. Introduction

Post-trauma chronic pain is one of the symptoms following traumatic brain injury (TBI). The overall prevalence of chronic pain in patients with a TBI varies depending on the cause of pain, but it is known to range from 22% to 95%, with higher prevalence in patients with a mild TBI [1,2]. After trauma, patients with a TBI often complain of chronic pain in areas without injuries. This chronic pain, called central pain characterized by neuropathic pain, is caused by damage to the central nervous system, including the brain and spinal cord [3,4]. Although some studies have been conducted to characterize the pathophysiology of central pain [4,5,6], the mechanisms underlying this type of pain post-TBI have not been clearly elucidated.

Based on previous studies, medications such as amitriptyline, an adrenergic antidepressant; lamotrigine, an antiepileptic drug; and GABAergic drugs have been widely used in clinical trials to treat central pain [7,8]. Aside from medications, recent studies have reported the positive therapeutic effect of repetitive transcranial magnetic stimulation (rTMS) for central pain [9,10]. rTMS is a noninvasive brain stimulation in which a changing magnetic field is used to cause an electric current at the brain through electromagnetic induction. Meanwhile, the spinothalamic tract (STT) is a major somatosensory tract that transmits sensory information upward to the somatosensory cortex of the postcentral gyrus past the spinal cord and thalamus, and the STT has been considered a plausible neural tract associated with central pain recently [11,12].

The development of diffusion-based tractography enabled the visual reconstruction of the STT three-dimensionally, and differences in left and right STT were reported in tractography derived from diffusion tensor imaging (DTI) in patients with central pain [11]. In addition, previous studies have reported the clinical effects of rTMS on central pain and the correlation between central pain and evaluation tools such as tractography derived from DTI, functional magnetic resonance imaging (MRI), single-photon emission computerized tomography and laser-evoked potential study [11,13,14]. However, few studies have examined the effect of rTMS in patients with mild TBI with central pain through changes in DTI-based metrics of STT before and after rTMS.

In this study, we performed rTMS in patients with a mild TBI and central pain to evaluate the pre-rTMS and post-rTMS outcomes, to confirm pain relief through self-report by patients, bedside sensory examination, as well as changes in DTI-based metrics of the STT. This study aimed to investigate the effect of rTMS on central pain symptoms in patients with mild TBI through diffusion-based tractography and DTI metrics of STT.

The institutional review board of the Jeonbuk National University approved this case report and waived the requirement for informed consent (IRB number CUH 2019-01-047).

## 2. Materials and Methods

### Case Report

This study presents four patients (39-year-old woman, 40-year-old man, 43-year-old woman, 45-year-old woman) with mild TBI who had no specific findings on conventional brain MRI. They were injured with their heads shaken by an in-car accident in which all vehicles rotated due to side collisions. Right after the car accident, all patients had a brief change in mental status (confusion, disorientation, or loss of memory) or loss of consciousness for <10 min, but had no neurologic sequelae such as motor deficits or cognitive impairment. All participants had no particular medical history before the accident. They commonly had pain starting 1 or 2 days after the car accident, which persisted despite continued treatment in other hospitals (Table 1).

**Figure 1:**
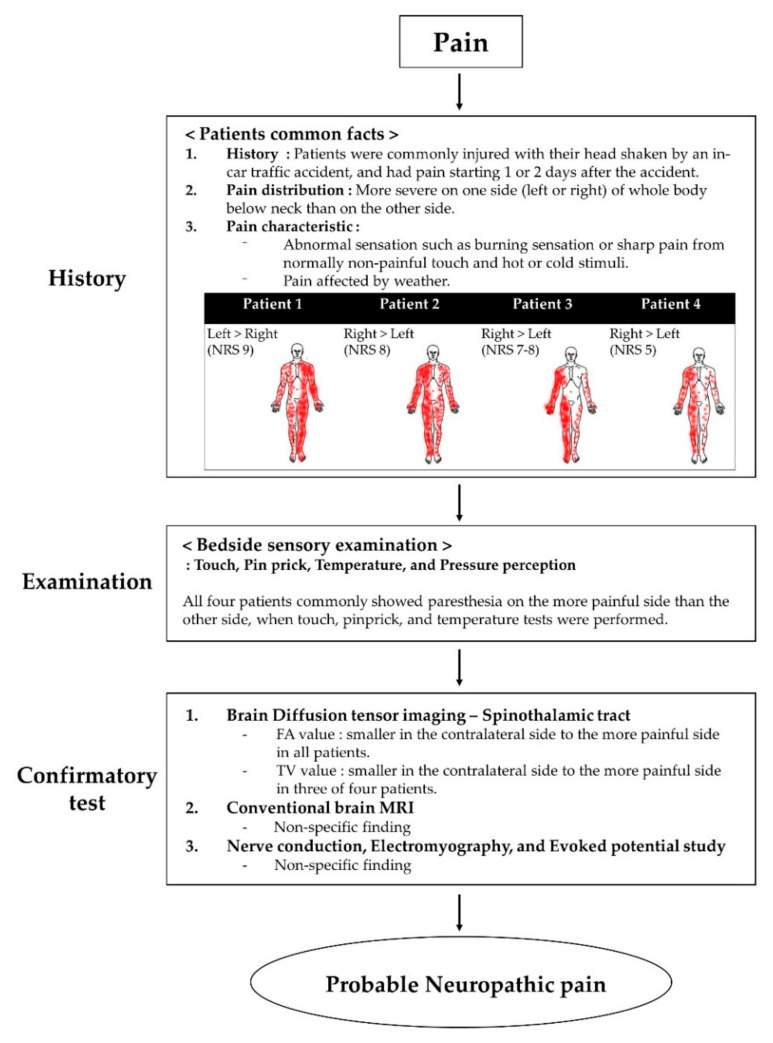
Flow chart of the grading system for neuropathic pain. Abbreviations: MRI, magnetic resonance imaging.

All four patients presented to our hospital with severe pain in the extremities and body. Their pain distribution was commonly more severe on one side (left or right) of the whole body below the neck. All patients expressed that the weather heavily affected their pain. They complained of abnormal sensations, such as burning or sharp pain, from normally non-painful touch and hot or cold stimuli. We asked the patients to express their pain intensity through a numerical rating scale (NRS), which is expressed as a score from 0 to 10, 0 means no pain, and 10 means maximum imaginable pain [15]. Moreover, we conducted bedside sensory examination, including non-painful stimuli such as touch, pinpricks, temperature, and pressure perception [16]. When touch, pinprick, and temperature tests were performed, all four patients commonly showed paresthesia on their more painful side. There was no difference in proprioception and vibration. The characteristics and distribution of pain and NRS are shown in Figure 1.

We conducted brain diffusion tensor imaging (DTI) to investigate the cause of pain. We acquired 49 contiguous slices parallel to the anterior commissure-posterior commissure line. Imaging parameters were as follows: acquisition time = approximately 3 min; acquisition matrix = 128 × 128; field of view = 220 mm × 220 mm; TR = 6600 ms; TE = 95 ms; parallel acquisition factor GRAPPA = 2; bandwidth = 1562 Hz/Px; EPI factor = 128; b = 1000 s/mm^2^; slice gap = 0 mm; and a slice thickness = 3 mm (acquired isotropic voxel size = 0.9 mm × 0.9 mm × 3.0 mm). After DTI, the spinothalamic tract (STT) was reconstructed to visualize the sensory fibers by applying the fiber tracking technique using the DTI studio software v.1.02 (CMRM, John Hopkins Medical Institute, Baltimore, MD, USA). Fiber tracking was performed with fractional anisotropy (FA) threshold of 0.20 and a tract turning angle of 60°. We performed reconstruction by selecting three regions of interest (ROIs) from the brain MRI axial slice: the seed ROI was set at the posterolateral medulla, the first target ROI was set at the point near the ventral posterior lateral nucleus of the thalamus, and the second target ROI was set at the primary somatosensory cortex [17]. Subsequently, we selected fiber bundles that passed through the seed and target ROI. Then, the FA and tract volume (TV) of the reconstructed STT were obtained, and the values of the left and right sides were compared [11,18,19]. The FA value represented the directionality of water diffusion and was quantified from 0 (completely isotropic diffusion) to 1 (completely anisotropic diffusion) [18,20]. The TV represents the number of voxels included in the neural tract [21,22]. Before rTMS, we obtained FA and TV of STT of patients and compared the side with severe pain to the side with mild pain.

To confirm the possibility of neural compromise, nerve conduction study (NCS), electromyography (EMG), and motor and sensory-evoked potential (MEP and SEP) studies were performed [23].

Recently, the guidelines have been reported to confirm that the pain complained of by patients is neuropathic pain. According to the guidelines of the diagnosis and grading for neuropathic pain, all patients had neuropathic pain corresponding to a “probable grade” because the confirmatory test was not evaluated fully [16,24] (Figure 1).

We applied rTMS to all patients as a treatment for central pain. The following rTMS protocol was employed based on a previous study: a series of 10 trains (one train contains 200 pulses, with a total of 2000 pulses) at a stimulation rate of 20 Hz and at an intensity of 80% of motor threshold using an 8-shaped coil on the M1 cortex (cortical site at which a single-pulse TMS evoked a contralateral motor-evoked potential of the maximal amplitude in a hand muscle) [9,10,14,25,26]. rTMS was applied once a day, five times a week, for three weeks. A total of 15 rTMS treatments was considered as a session. All the patients applied rTMS on the M1 cortex contralateral to the more painful side. During the rTMS treatment, there was no change in medications and other interventions.

After the end of the rTMS, follow-up NRS scale of pain and bedside sensory examination of patients were checked between 3 and 6 days, and follow-up DTI, NCS, EMG, MEP and SEP were performed within 3 months.

## 3. Results

Of the four patients, patients 1 and 2 reported high satisfaction with pain relief after the first session; thus, they underwent an additional session on the side opposite to the first session. Bedside sensory examination, including non-painful stimuli such as touch, pinpricks, and temperature tests, showed improvement in paresthesia, like a burning or sharp sensation, in all patients. Moreover, pain relief was reported by all patients, with the NRS decreased from 9 to 4 in patient 1, from NRS 8 to 5 in patient 2, from NRS 7–8 to 4 in patient 3, from 5 to 1 in patient 4 (Table 2).

**Figure 2:**
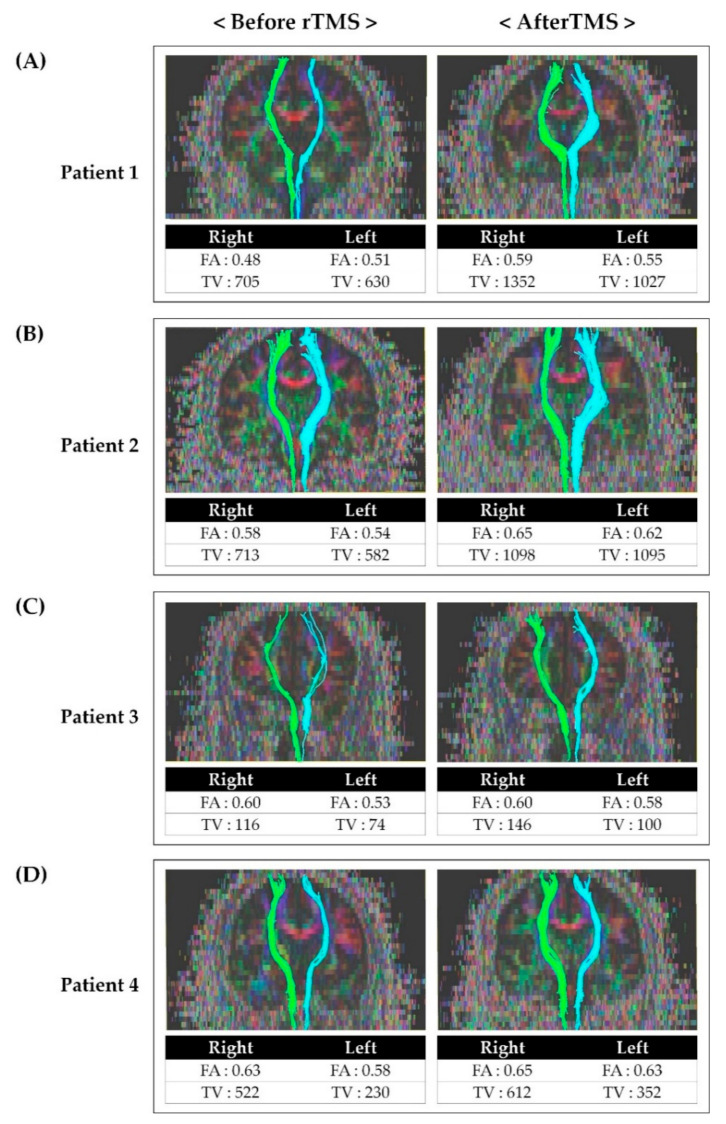
Tractography for the spinothalamic tract in patients before and after repetitive transcranial magnetic stimulation: (**A**) Tractography for STT in patient 1 before and after rTMS. (**B**) Tractography for STT in patient 2 before and after rTMS. (**C**) Tractography for STT in patient 3 before and after rTMS. (**D**) Tractography for STT in patient 4 before and after rTMS. After rTMS, FA and TV values had increased compared to the value before rTMS. Abbreviations: rTMS, repetitive transcranial magnetic stimulation; FA, fractional anisotropy; TV, tract volume; STT, spinothalamic tract.

Before rTMS, the FA and TV values of the contralateral STT on the more painful side were generally lower than on the other side in all patients. The FA and TV values after rTMS was applied were increased compared to the results before rTMS (Figure 2).

The patients had nonspecific findings of NCS, EMG, MEP, and SEP before and after rTMS. In all patients, no adverse effects such as headaches or dizziness from applying rTMS were reported.

## 4. Discussion

Central pain after TBI has a relatively late onset, ranging from several weeks to several months. Pain occurs in various locations, but the central pain of supraspinal causes is known to occur mainly in the hemi-side, or the hemi-side generally shows worse conditions. Central pain is generally characterized by neuropathic pain, which is a stimulation-independent pain similar to pricking, throbbing, and burning sensations, accompanied by responses from physical examination including allodynia, hyperpathia, and paresthesia [3,6]. Patients in this study commonly had asymmetric severe neuropathic pain and abnormal perception of temperature sensation, with worse conditions on either the left or right side. Many patients with central pain showed an autonomic dysfunction, and the pain was increased by physical and emotional stress and alleviated by relaxation [27]. Moreover, the patients in this study commonly had asymmetric severe neuropathic pain and abnormal perception of temperature sensation, with worse conditions on either the left or right side. Related studies of central poststroke pain (CPSP) have also observed similar aspects of pain and abnormalities, and the STT was considered the most plausible neural tract which is responsible for the pathogenesis of CPSP [12,28].

Two common pathophysiological processes are assumed to be the cause of painful overreaction for central pain itself and for somatic stimulation, which accompanies central pain. First, the hypothesis of “irritative lesions” suggests that hyperactive cells at or near the lesion increased the activity in the normal nociceptive pathway. Second, the hypothesis of “denervation or hypersensitivity” suggests that although neurons are far from the lesion, as the nociceptive processing pathways have lost their normal synaptic input, they become hyperactive and hypersensitive [29]. The two hypotheses are not mutually exclusive but rather compatible, and both hypothetical processes can contribute in varying degrees to the pathophysiology of patients who complained of central pain. Therefore, neurological examinations and imaging tests are needed to examine the integrity of ascending tracts, such as the STT, when treating patients with mild TBI and central pain.

Recently, visualization of the STT to reflect the damage at the thalamic level has become possible from the development of tractography derived from DTI. There has been a report of left- and right-side STT differences in tractography of patients with central pain [11,30]. The results of this study suggest that the group of patients with central pain generally had lower FA and TV values on the contralateral side of the STT with worse pain than those found in the STT of the other side. The STT was considered the most plausible neural tract responsible for the pathogenesis of central poststroke pain, which was consistent with the observed similar aspects of pain and abnormalities in this study [12].

Studies on the effect of rTMS on central pain and the association between central pain and STT have been reported. The mechanisms of rTMS to the motor cortex for neuropathic pain can be explained by a reduction in pain-related thalamic hyperactivity, regional cerebral blood flow changes, activation of descending pathways, and restoration of intracortical inhibition. However, consensus on the exact mechanism has not yet been achieved [5,10,31]. Although there are reports of the effect of rTMS on central pain, no studies have confirmed the effect of rTMS on central pain by comparing STT before and after rTMS. Therefore, assuming that pain reduction after rTMS may be related to the alleviation of STT problems, we performed follow-up DTI after rTMS (Figure 2). FA values indicate the directionality and integrity of white matter microstructures, and the reduced FA values are associated with impaired integration of the neural tract [18,20,32]. Moreover, the TV value represents the number of voxels included in the neural tract [32]. Therefore, lower FA and TV values more severe impairment of STT. After rTMS, it was observed that both FA and TV values had increased compared to those before rTMS. In addition, we also confirmed that the patients’ NRS and bedside sensory examination results improved as well. Considering the increase in FA and TV and improvement of symptoms after rTMS, rTMS is thought to have induced inhibition of hypersensitivity of STT and affected the integration of STT.

We enrolled patients with probable neuropathic pain via the neuropathic pain grading system and investigated the efficacy of rTMS in central pain relief through self-report by patients, bedside sensory examination, as well as recovery of STT using tractography. Evaluating the integrity of STT through diffusion-based tractography may be an appropriate assessment option for assessing and following up on the degree of central pain. Additional randomized controlled trials will be needed in the future to more clearly verify the results of this study.

## Figures and Tables

**Table 1:** Baseline demographic and clinical characteristics of patients.

	Patient 1	Patient 2	Patient 3	Patient 4
Age	39	40	43	45
Sex	Female	Male	Female	Female
Time of pain onset	1 day	1 day	2 days	1 day
Vector	In-car accident—vehicle rotated due to side collision			

**Table 2:** The numeric rating scale of patients before and after rTMS.

	Before rTMS	After rTMS
Patient 1	9	4
Patient 2	8	5
Patient 3	7–8	4
Patient 4	5	1

rTMS: repetitive transcranial magnetic stimulation.
